# A New Orientation Detection Method for Tilting Insulators Incorporating Angle Regression and Priori Constraints

**DOI:** 10.3390/s22249773

**Published:** 2022-12-13

**Authors:** Jianli Zhao, Liangshuai Liu, Ze Chen, Yanpeng Ji, Haiyan Feng

**Affiliations:** Electric Power Research Institute, State Grid Hebei Electric Power Co., Ltd., Shijiazhuang 050017, China

**Keywords:** tilting insulator, orientation detection, angle regression, prior constraint

## Abstract

The accurate detection of insulators is an important prerequisite for insulator fault diagnosis. To solve the problem of background interference and overlap caused by the axis-aligned bounding boxes in the tilting insulator detection tasks, we construct an improved detection architecture according to the scale and tilt features of the insulators from several perspectives, such as bounding box representation, loss function, and anchor box construction. A new orientation detection method for tilting insulators based on angle regression and priori constraints is put forward in this paper. Ablation tests and comparative validation tests were conducted on a self-built aerial insulator image dataset. The results show that the detection accuracy of our model was increased by 7.98% compared with that of the baseline, and the overall detection accuracy reached 82.33%. Moreover, the detection effect of our method was better than that of the YOLOv5 detection model and other orientation detection models. Our model provides a new idea for the accurate orientation detection of insulators.

## 1. Introduction

As an essential component of transmission lines, insulators undertake the functions of electrical insulation and structural support [1]. Under multiple impacts of high voltage, mechanical stress, and harsh environment, insulators are prone to defects such as fouling, flashing, breakage, and string dropping. In this case, the fast and accurate detection of insulators and their defects has become an essential task to ensure the safety of transmission lines [2,3]. Many relevant studies focused on the accuracy and speed of detection [4,5], but an equally important issue, how to accurately recognize those tilting insulators, still needs exploring.

Insulator detection methods [6,7,8,9,10,11] can be roughly classified into two categories: digital-image-processing-based methods and deep-learning-based methods. The insulator contamination detection method proposed by Xun et al. [6] is a typical method using digital-image processing technology. It improves the watershed algorithm by similar-region fusion and minimization segmentation and effectively avoids the over-segmentation phenomenon. Zhai et al. [7] introduced airspace morphological consistency features to obtain high-accuracy insulator pinpointing in the insulator detection task. Zhang et al. [8] generated feature sequences by various texture extraction methods and achieved good insulator-defect detection results. In the absence of background interference, insulator detection methods based on digital-image processing have high detection accuracy, but they rely too much on artificially designed features and have poor robustness, which makes them struggle to handle aerial images taken by unmanned aerial vehicles (UAV) with complex background environments and small insulator targets. With the development of UAV image-acquisition technology and object detection technology in electric power field, deep learning based insulator and defect detection methods have been widely investigated. For example, Wang et al. [9] combined a two-stage insulation anomaly detection model with a few-sample learning method to achieve high-precision defect detection. Following the two-stage detection idea of combining the target detection task with the semantic segmentation task, Ling et al. [10] proposed a lightweight and high-precision insulator detection method. Li et al. [11] applied the YOLOv5 series model to the insulator detection task and achieved a detection accuracy of up to 96% and a detection speed of 42 images per second on the self-built insulator detection dataset, proving the superiority of the YOLOv5 series model.

Currently, great breakthroughs have been made in the study on the insulator detection of aerial insulator images. However, most of the methods are the simple migration of generic object detection to insulator detection. In some complex conditions such as dense mutual occlusion and dense distribution, which often result in unnecessary background noise and overlap, the insulator detection effect is not ideal, and sometimes there even exists a phenomenon of missing detection [12]. In aerial images shot by UAVs, insulators appear with different aspect ratios and tilt angles, while the general object detection models cannot fully utilize the scale and tilt features. When detecting insulator overlap, if the axis-aligned bounding boxes are too close to each other, the non-maximum suppression algorithm often fails, resulting in missing detection.

In this paper, we apply the YOLOv5 model to the insulator detection field and propose a tilting-insulator detection model based on angle regression and prior constraint of scale (RAPC-YOLO). In particular, we firstly introduce the angle regression loss in the loss function and combine the oriented bounding box with the YOLOv5 object detection model, thus effectively improving the tilting-insulator detection effect. Then, the anchor box parameters of the detection model are analyzed and optimized by the clustering algorithm according to the scale features of the insulators. Last but not least, we introduce a rotational uncertainty function to guide the learning of the angle regression loss according to the angle distribution of the insulator’s oriented bounding box, so as to improve the robustness of the model to the tilting angle.

## 2. Materials and Methods

**Figure 1:**
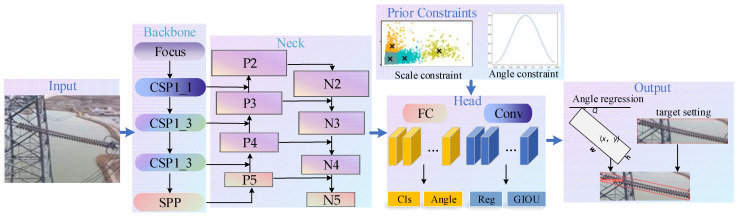
Architecture of RAPC-YOLO.

In this section, an overview of our proposed method is given. The architecture of the tilting insulator detection model is shown in Figure 1. The detailed optimization adjustments on the YOLOv5 network is given in Section 2.1. After that, a specific method to implement the scale priori constraint is presented in Section 2.2. Finally, the influence of the tilting angle is analyzed, and a method to obtain the angle priori constraints is put forward in Section 2.3.

### 2.1. YOLOv5-Orientation Model

YOLOv5 is the fifth generation of the You Only Look Once (YOLO) series of single-stage detection models, and it has become one of the most popular baseline models in the field of target detection. To obtain the reliable detection of tilting insulators, we introduce the angle parameter to the axis-aligned bounding box to form an oriented bounding box adapted to the tilt characteristics of insulators. Then an angle regression loss is introduced into the loss function, so as to obtain a new YOLOv5-Orientation model adapted to the tilting-insulator detection task using loss descent learning.

The oriented bounding box parameter of the YOLOv5-Orientation model is defined as [x,y,w,h,θ], where (x,y) is the normalized centroid coordinates, *w* is the normalized short edge length, *h* is the normalized long edge length, and θ is the normalized tilt angle. The normalized tilt angle θ is derived from Equation (1).

(1) $$\theta =\frac{Q}{90}$$

where *Q* is the tilt angle.

**Figure 2:**
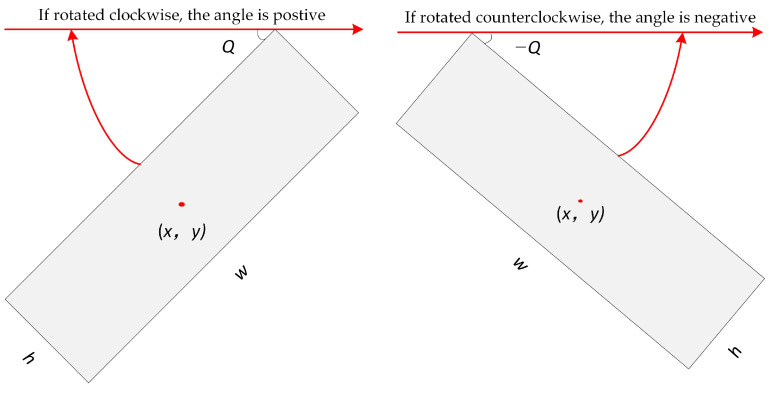
Schematic diagram of the oriented bounding box.

As shown in Figure 2, *Q* is the minimum angle required for the long side *w* of the rectangular box to coincide with the *x*-axis. If the rotation is clockwise, the tilt angle is positive; otherwise the tilt angle is negative. Thus, the value range of *Q* is [−90°, 90°].

An angle regression loss based on the localization loss of the YOLOv5 model is introduced, consisting of the generalized intersection over union (GIOU) [13] loss and the smoothing loss [14], as expressed by Equation (2).

(2) $$L_{reg}(o,l,g)=\sum_{i}\sum_{m\in S}o_{ij}SL_{GIOU}(l_{i}^{m}-g_{j}^{\Delta m})+\sum_{i}o_{ij}\theta L_{1}(l_{i}^{\theta }-g_{j}^{\Delta \theta })$$

where *o* denotes the label, *l* denotes the predicted oriented bounding box, *g* denotes the real oriented bounding box, *o_ij_* is a binary variable and denotes the degree of matching between the label of the *i*-th default box and the label of the *j-*th real box, *S* is the set of parameters {*x,y,w,h*}, $g_{j}^{\Delta m}$ is the offset of parameters {*x,y,w,h*}, $g_{j}^{\Delta \theta }$ is the introduced angle offset, and the offset calculation formula is shown in Equation (3).

(3) $$\left\{\begin{array}{l} g_{j}^{\Delta x}=(g_{j}^{x}-d_{i}^{x})/d_{i}^{w}\\ g_{j}^{\Delta w}=\mathrm{lg}(\frac{g_{j}^{w}}{d_{i}^{w}})\\ g_{j}^{\Delta y}=(g_{j}^{y}-d_{i}^{y})/d_{i}^{h}\\ g_{j}^{\Delta h}=\mathrm{lg}(\frac{g_{j}^{h}}{d_{i}^{h}})\\ g_{j}^{\Delta \theta }=(g_{j}^{\theta }-d_{i}^{\theta }) \end{array}\right.$$

The center coordinate offset ${(g}_{j}^{\Delta x}{,g}_{j}^{\Delta y})$ is the normalized value of the difference between the center coordinates of the real rectangular box *g* and the default rectangular box *d*. The long side offset $g_{j}^{\Delta w}$ and the short side offset $g_{j}^{\Delta h}$ are the logarithm of the corresponding side-length ratio between the real rectangular box *g* and the default rectangular box *d*. The above four offsets are regressed by GIOU loss, and the formula is shown in Equation (4). The angle offset $g_{j}^{\Delta \theta }$ is the difference between the tilt angle $g_{j}^{\theta }$ of the real rectangular box *g* and the tilt angle $d_{j}^{\theta }$ of the default rectangular box *d*, and it is regressed by *L*_1_-smoothing loss. The formula is shown in Equation (5).

(4) $$L_{GIOU}=1-GIOU$$

(5) $$smooth_{L_{1}}(m)=\left\{\begin{array}{ll} 0.5\;m^{2} & if\;|m|<1\\ |m|-0.5 & otherwise \end{array}\right.$$

### 2.2. Scale Priori Constraints

Motivated by the optimization need of the initial anchor-box aspect ratio and number, the scale priori constraints are put forward. The preset parameters of the anchor boxes of the YOLOv5-Orientation model are extracted from the public dataset, and they do not match the insulator scale features. Therefore, in this paper, the K-means [15] clustering algorithm is used to cluster and analyze the scale parameters of each labeled oriented bounding box in the insulator dataset. The width–height ratio, size, and number of the optimized anchor box are used as the new anchor-box preset parameters. The specific implementation process is shown in Algorithm 1.
**Algorithm 1** Overall process of anchor-box scale clustering**Input:**   The scale parameters of labeling box in the dataset, the maximum number of iterations *I* **Processing:**   **for***m* = 1; *m* ≤ *I*; *m*++ **do** The set of parameter samples obtained from the dataset T = ${\{t}_{i}{|t}_{i}\in R^{v},i=1,2,3,\ldots M\}$$,\;t_{i}$ is a single sample, *M* is the number of labeled boxes, *v* = 2 is the parameter dimension, which is the width and height parameter, respectively.Randomly initialize K samples as clustering centers to form the set of clustering centers $\;C\left(I\right){=\{c}_{j}{|c}_{j}\in R^{v}\}$$,\;c_{j}$ is a single clustering center, *I* is the number of iterations, and its initial value is 1.Calculate the distance between each sample $t_{i}$ of the sample set *T* and each cluster center $c_{j}$ in $C\left(I\right)$ according to the Euclidean distance formula $d(t_{i},c_{j})=\sqrt{{\left(t_{i}-c_{j}\right)}^{2}}$ and merge each sample $t_{i}$ into the cluster center $c_{j}$ with the smallest Euclidean distance by the size of the Euclidean distance, i.e.,$T_{j}{=\{t}_{i}{|t}_{i}\subseteq c_{j}\}$.For each cluster $T_{j}$ take the sample $t_{i}$ belonging to it and calculate the new cluster center $\tilde{c_{j}}$ according to Equation $\tilde{c_{j}}=\sum_{t_{i}\in T_{j}}t_{i}/n$, and form the set of cluster centers $C\left(I+1\right)$ from the new cluster centers $\tilde{c_{j}}$.
   **if**
$C\left(I+1\right)=C\left(I\right):$    **end for** **Output:** The width, height, and number parameters obtained by clustering

As an important hyperparameter of the K-means algorithm, *K* directly affects the clustering quality. In order to obtain the clustering results with high intra-cluster similarity and low inter-cluster similarity, CH is selected to measure the effects of different K-values on clustering quality, so as to obtain the best anchor-box preset parameters, calculated by Equation (6).

(6) $$CH(K)=\frac{traceB/\left(K-1\right)}{traceW/\left(N-K\right)}$$

where, *traceB* denotes the trace of the inter-cluster dispersion matrix, *K* denotes the number of clustering centers, *traceW* denotes the trace of the intra-cluster dispersion matrix, *N* denotes the total number of records, and *CH* is proportional to the clustering quality.

### 2.3. Angle Priori Constraint

**Figure 3:**
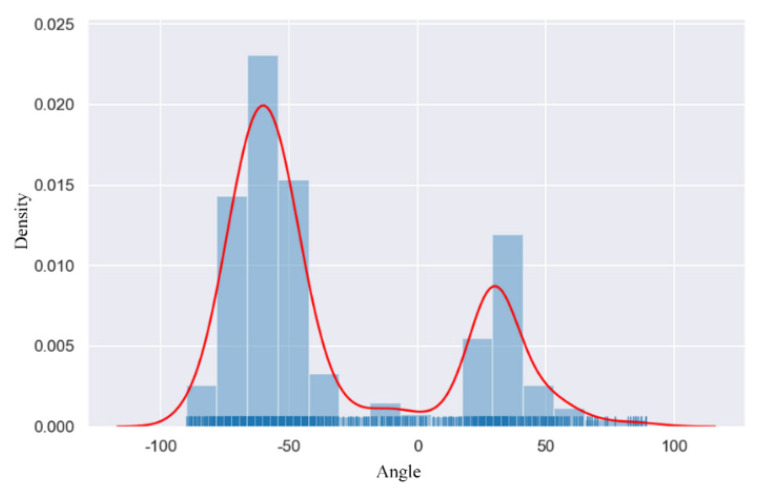
Probability density distribution of tilt angle in the insulator dataset.

Studies [16,17,18] have shown that balanced sample distribution has a significant impact on detection performance. Therefore, the analysis of the insulator-tilting-angle samples is necessary, and the optimization of the unbalanced sample distribution can improve the robustness of the model to the tilt angle. Figure 3 shows the frequency distribution and probability density distribution of the tilt angle of the annotated boxes in the insulator dataset. The light blue histogram is the angular frequency, and the red curve is the fitted probability density.

It can be found that the overall distribution of the tilt angles is uneven. A large number of the tilt angles of these samples are concentrated around −60° and 30°. To reduce the effect of the uneven distribution of the tilt angle on the angle regression, we introduce a rotational uncertainty function [16] as a threshold function to control the regression loss, so as to obtain the angle prior constraint.

**Figure 4:**
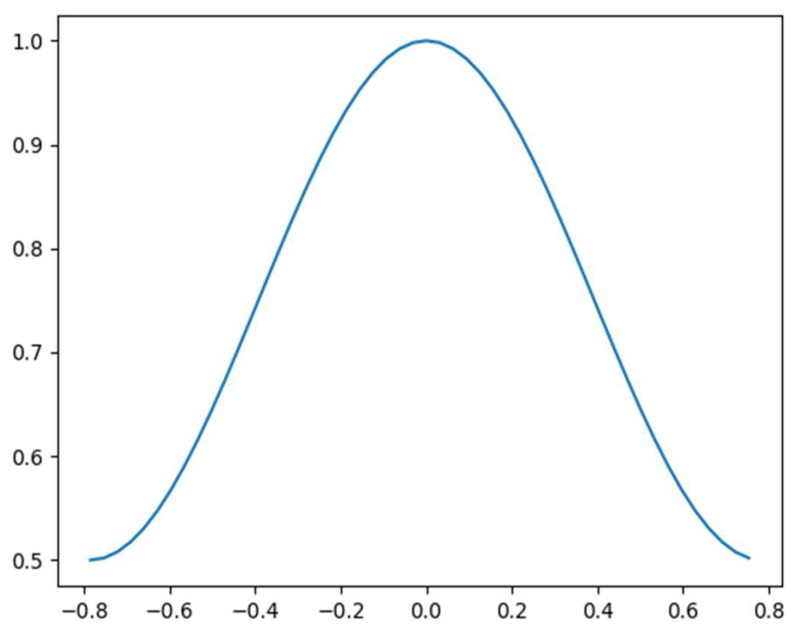
Visualization of rotational uncertainty function.

The formula of the rotational uncertainty function D(θ) is shown in Equation (7), where θ is the tilt angle, and δ is the angular hyperparameter when $D\left(\theta \right)=0.5$. The visualization graph of the rotational uncertainty function is shown in Figure 4.

(7) $$D(\theta )=\max(0.5,1+\frac{1-\cos(4\theta )}{2\cos(4\delta )-2})$$

This function maps the tilt angle θ to the GIOU threshold and then controls the regression loss calculation by the GIOU threshold. In this way, the semantic features learned by the model in the interval with more distribution of tilt-angle samples can be migrated to the interval with less distribution of tilt-angle samples, so as to assist their detection. Herein, the GIOU threshold is set to 0.5 in reference to the threshold of anchor matching in the standard object-detection architecture.

## 3. Test Results & Analysis

### 3.1. Test Data and Parameter Settings

The test dataset consists of insulator images taken by UAVs, including 1754 aerial images of insulators. Most images contain tilting insulators. About 1404 aerial-insulator images were randomly selected from the dataset to form a training set, while the remaining for a test set, and the ratio of the training set to the test set was 4:1.

In this paper, average precision (AP) is chosen as the test evaluation metric. It can reflect the comprehensive accuracy of each category and is derived by integrating the P–R curves constructed from recall and precision, as shown in Equation (8).

(8) $$AP_{m}=\sum_{m}\int_{0}^{1}P(r)d(r)$$

where *P*(*r*) is the curve with recall as the independent variable and precision as the dependent variable; *m* is the GIOU threshold. Precision is the proportion of correctly predicted boxes, and recall is the proportion of predicted boxes among all of the real boxes. As shown in Equations (9) and (10), *TP* denotes the number of correctly detected targets, *FP* denotes the number of incorrectly detected targets, and *FN* denotes the number of unpredicted real boxes.

(9) $$\mathrm{Precision}=\frac{TP}{TP+FP}$$

(10) $$\mathrm{Recall}=\frac{TP}{TP+FN}$$

To verify the effectiveness of our proposed method for the tilting-insulator detection task, we adopt AP_50_ as the basic evaluation index and also select AP_50~95_ as another evaluation index, which is more demanding for inspection.

The tests were conducted on an Ubuntu 18.04 operating system. The memory is 32GB; the graphics card is Nvidia GeForce RTX2080Ti, and the processor model Intel Core i9 10850K. Our building and training test work is conducted under Pytorch 1.8, CUDA 11.0. The initial learning rate used in the model is 0.01; the learning-rate decay strategy is exponential decay; the weight decay is set to 0.0005; the number of training rounds is set to 100, and the batch parameters is set to 8.

### 3.2. Scale Priori Constraint Analysis

**Figure 5:**
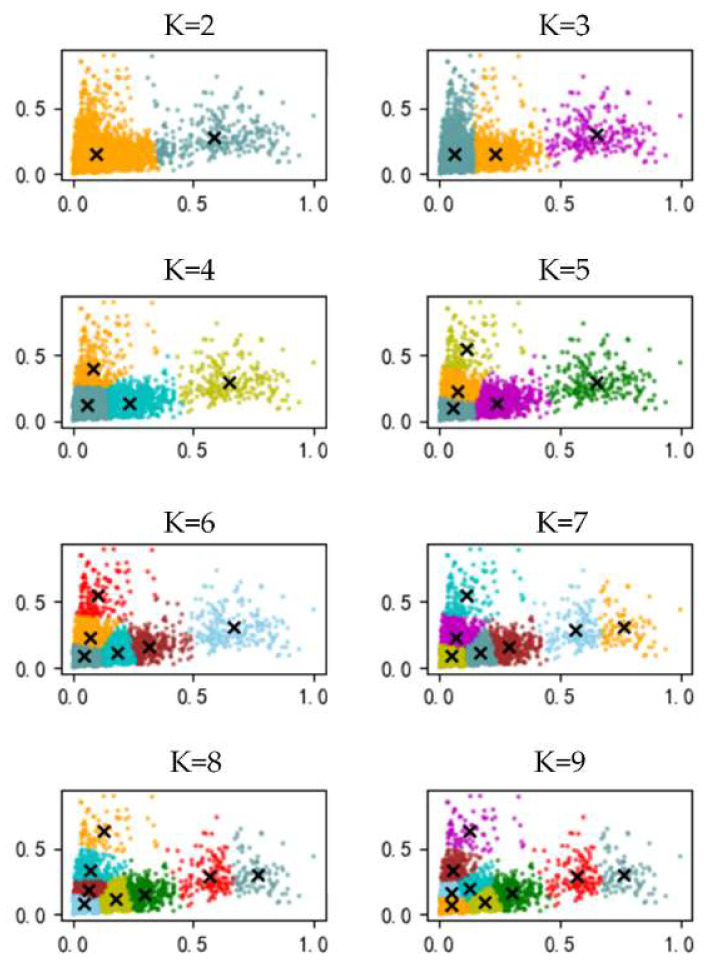
Aspect ratio clustering result.

In this test, the width–height ratios of 2211 insulator annotation boxes were extracted from 1404 training sets of aerial images of insulators and were used as input. The annotation boxes were clustered and analyzed by controlling the cluster center number to search for the optimal width–height ratio, size, and number of anchor boxes. The width–height ratio clustering results are shown in Figure 5, where the horizontal and vertical coordinates represent the normalized width and normalized height of the insulator annotation boxes, respectively. The cross symbols in the figure represent the clustering centroids, and different clusters are distinguished by different colors. It can be seen from Figure 5 that the insulator dataset has a wide range of width–height ratio distribution, obvious differences in the width–height ratio between samples, and a large-scale span, etc. From the clustering results, it can be seen that the width–height ratio of the insulator dataset clustering center is roughly in the range of [0.3, 3].

Under the condition of different clustering center numbers, the clustering results of normalized width and normalized height were used as input to calculate the corresponding *CH*s, and the results are listed in Table 1.

From Table 1, it can be seen that *CH* corresponding to the number of clustering centers of four is the largest, i.e., the best clustering effect. At this time, the width–height ratio interval of [0.38, 2.52] derived from clustering is obviously beyond the preset anchor-box width–height ratio interval of [0.5, 2]. Therefore, the initial anchor-box aspect ratio is set to (1:3, 1:1, 3:1), and its corresponding aspect ratio interval [0.33, 3.0] covers the insulator aspect-ratio interval derived from the clustering, which is a good fit for the insulator size c features. Meanwhile, the center point size can be deduced from the coordinates of the center of clustering in an interval of 80^2^–68^2^. In order to match the insulator annotation-box size distribution, the anchor-box size is set to [2, 4, 8, 16, 32]. It can thus cover the original image size of 16^2^–256^2^ in the case of the minimum perceptual field and the original image size of 64^2^–1024^2^ in the case of the maximum perceptual field that contains the insulator annotation-box size distribution in different perceptual fields.

### 3.3. Ablation Test and Comparison Test

In order to evaluate the performance of the improved method, ablation tests were conducted, and the results are shown in Table 2. The YOLOv5-Orientation model was selected as the baseline model; the YOLOv5- Orientation model with the introduction of the scale priori constraints is Improved Model 1; the YOLOv5-Orientation model with the introduction of the angle priori constraints is Improved Model 2, and the RAPC-YOLO is the model proposed in this paper.

As can be seen from Table 2, when GIOU was taken as 50%, the AP value of the baseline model on the tilting insulator dataset was only 74.35%. Compared to the baseline, the detection accuracies of Improved Model 1, Improved Model 2, and the RCPA-YOLO model were increased by 4.88%, 3.57%, and 7.98%, respectively. When GIOU was in the range of 50% to 95%, the AP value of the baseline model in the tilting-insulator dataset was only 34.21%. Compared with the baseline, the detection accuracy of Improved Model 1 was increased by 9.18%, and that of Improved Model 2 was increased by 5.53%. For the RCPA-YOLO model, the accuracy reached 51.51%, an increment of 17.3% compared with the baseline, a quite significant improvement.

The ablation tests show that both the scale priori constraints and the angle priori constraints can effectively improve the detection accuracy of the baseline. The priori constraint method is more effective at high GIOU thresholds, indicating that the priori constraint method can help to accurately position the tilting insulators.

**Figure 6:**
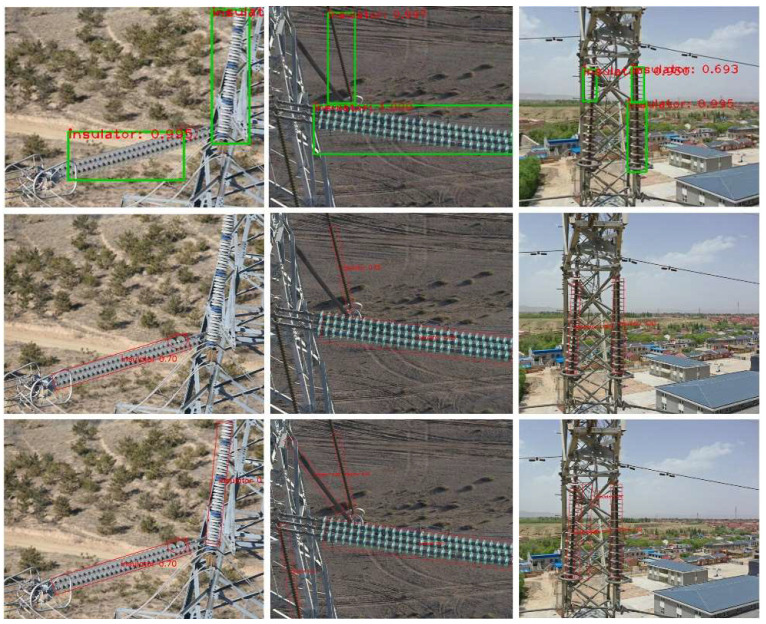
Comparison of detection results among our model and other detection models.

The insulator detection results of the three models are demonstrated in Figure 6, where rows 1, 2, and 3 are the visualized detection results of the YOLOv5 model, the YOLOv5-Orientation model, and the RAPC-YOLO model, respectively. It can be seen from the figure that the YOLOv5 model has problems such as the misdetection of obscured insulators and the incomplete overlapping of detection bounding boxes. Besides, its detection bounding boxes are positive rectangles containing a large amount of complex background information. Although the YOLOv5-Orientation model can detect some tilting insulators, there still exist some problems such as misdetection or false detection for insulators with wide tilt angles. In contrast, our RAPC-YOLO model achieves a better detection effect for tilting insulators. The additional priori constraint module makes the oriented bounding box accurately surround the tilting insulators and realizes positioning with better precision.

Xue et al. proposed an oriented object detection model R3Det [19], based on a feature pyramid network (FPN). X. Yang et al. proposed another oriented object detection model SCRDet [20] for remotely sensed small-target object detection. These two models can be used to detect objects with arbitrary angles. In order to verify the effectiveness of the tilting-insulator detection model RAPC-YOLO, a detection performance comparison of several oriented object detection models and the RAPC-YOLO model was made in this paper under the same test conditions. The results are shown in Table 3. As can be seen, the detection accuracy of the RAPC-YOLO model in the tilting-insulator detection task is superior over other oriented object detection models. Moreover, the detection performance of the RAPC-YOLO model is more remarkable with higher intersection-over-union (IOU) thresholds, which further verifies that the priori constraint method helps to improve the detection accuracy of the model.

**Figure 7:**
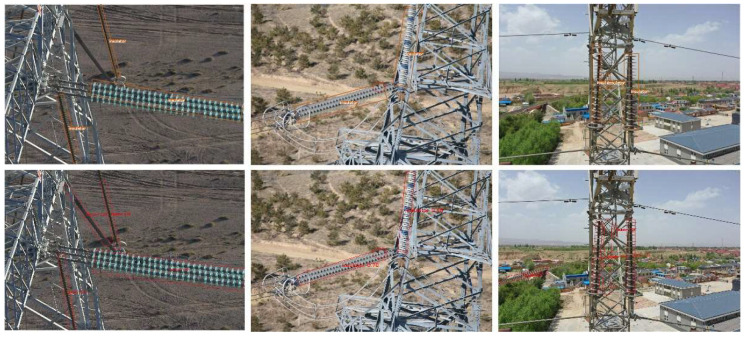
Comparison of the detection results of our model and SCRDet++.

In addition, we selectthe SCRDet++ model with the highest accuracy to carry out a visual comparative analysis with the model proposed in this paper. The results are shown in Figure 7. It can be seen from the figure that the SCRDet++ model has the problem of misdetection, especially for the shading insulator and the insulators that are relatively close to each other. In contrast, these problems are better solved in the model proposed in this paper.

## 4. Conclusions

In this paper, we propose a RAPC-YOLO model, a new orientation detection method for tilting insulators by fusing angle regression with prior constraints. We used an oriented bounding box, angle regression loss, and rotational uncertainty function to learn the tilting features of insulators. Furthermore, we applied a clustering algorithm to learn the insulator aspect ratio and size distribution. Ablation tests and comparison tests show that our RAPC-YOLO model is an effective architecture for tilting-insulator detection tasks. In our RAPC-YOLO model, the oriented bounding box fitting the insulator edges are generated, and thus the detection effect is significantly improved compared to the baseline model, especially in the aspects of false detection and anchor-box mismatch. In addition, the results show that RAPC-YOLO is superior over other models in detection accuracy. In the future, research such as insulator-defect detection will be further carried out on the basis of the proposed RAPC-YOLO.

## Figures and Tables

**Table 1:** Clustering results under different cluster centers.

K	Center Coordinates	CH
2	[80, 134] [342, 134]	5545.599
3	[81, 90] [90, 225] [347, 133]	6018.386
4	[71, 92] [88, 226] [277, 108] [430, 167]	6624.259
5	[67, 70] [430, 167] [266, 102] [100, 282] [415, 163]	6363.596
6	[65, 68] [76, 155] [89, 272] [246, 103] [391, 130] [447, 354]	6544.842
7	[56, 70] [66, 161] [173, 97] [96, 275] [302, 117] [431, 136] [445, 375]	6325.461
8	[59, 56] [57, 126] [82, 200] [177, 95] [94, 305] [302, 118] [431, 136] [445, 375]	6313.174
9	[51, 69] [63, 156] [158, 73] [75, 278] [293, 88] [171, 184] [442, 123] [340, 179] [461, 389]	6183.416

**Table 2:** Ablation test result.

	Scale Constraints	Angle Constraint	AP_50_	AP_50–95_
Baseline			74.35%	34.21%
Improved Model 1	√		77.92%	39.74%
Improved Model 2		√	79.23%	43.39%
RCPA-YOLO	√	√	82.33%	51.51%

**Table 3:** Performance comparison of different detection models.

Model	AP_50_	AP_50–95_
R3Det	54.29%	14.68%
SCRDet	71.64%	35.54%
SCRDet++ [21]	73.7%	40.83%
RAPC-YOLO	82.33%	51.51%

## Data Availability

Not applicable.

## Abbreviations

The following abbreviations are used in this manuscript:

UAV	Unmanned Aerial Vehicle
RAPC-YOLO	Regression of Angle and Prior Constraints YOLO
YOLO	You Only Look Once
GIOU	Generalized Intersection Over Union
CPLID	Chinese Power Line Insulator Dataset
AP	Average Precision
IOU	Intersection Over Union
FPN	Feature Pyramid Network
